# Checkpoint Signaling, Base Excision Repair, and PARP Promote Survival of Colon Cancer Cells Treated with 5-Fluorodeoxyuridine but Not 5-Fluorouracil

**DOI:** 10.1371/journal.pone.0028862

**Published:** 2011-12-15

**Authors:** Liyi Geng, Amelia M. Huehls, Jill M. Wagner, Catherine J. Huntoon, Larry M. Karnitz

**Affiliations:** 1 Division of Oncology Research, Mayo Clinic, College of Medicine, Rochester, Minnesota, United States of America; 2 Department of Molecular Pharmacology and Experimental Therapeutics, Mayo Clinic, College of Medicine, Rochester, Minnesota, United States of America; 3 Department of Radiation Oncology, Mayo Clinic, College of Medicine, Rochester, Minnesota, United States of America; Vanderbilt University Medical Center, United States of America

## Abstract

The fluoropyrimidines 5-fluorouracil (5-FU) and FdUrd (5-fluorodeoxyuridine; floxuridine) are the backbone of chemotherapy regimens for colon cancer and other tumors. Despite their widespread use, it remains unclear how these agents kill tumor cells. Here, we have analyzed the checkpoint and DNA repair pathways that affect colon tumor responses to 5-FU and FdUrd. These studies demonstrate that both FdUrd and 5-FU activate the ATR and ATM checkpoint signaling pathways, indicating that they cause genotoxic damage. Notably, however, depletion of ATM or ATR does not sensitize colon cancer cells to 5-FU, whereas these checkpoint pathways promote the survival of cells treated with FdUrd, suggesting that FdUrd exerts cytotoxicity by disrupting DNA replication and/or inducing DNA damage, whereas 5-FU does not. We also found that disabling the base excision (BER) repair pathway by depleting XRCC1 or APE1 sensitized colon cancer cells to FdUrd but not 5-FU. Consistent with a role for the BER pathway, we show that small molecule poly(ADP-ribose) polymerase 1/2 (PARP) inhibitors, AZD2281 and ABT-888, remarkably sensitized both mismatch repair (MMR)-proficient and -deficient colon cancer cell lines to FdUrd but not to 5-FU. Taken together, these studies demonstrate that the roles of genotoxin-induced checkpoint signaling and DNA repair differ significantly for these agents and also suggest a novel approach to colon cancer therapy in which FdUrd is combined with a small molecule PARP inhibitor.

## Introduction

**Figure 1:**
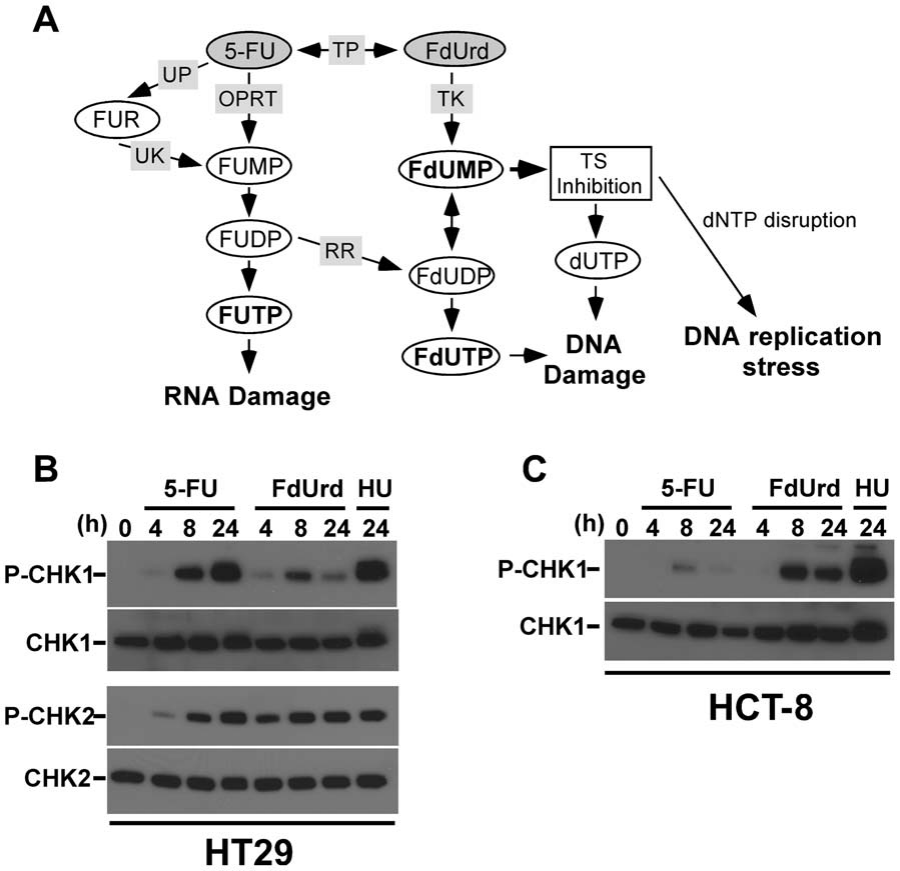
5-FU and FdUrd activate the ATR and ATM checkpoint signaling pathways. (A) Metabolism of 5-FU and FdUrd. (B, C) HT29 (B) and HCT-8 (C) cells were treated with 5-FU (80 µM, HT29 cells; 200 µM HCT-8 cells), FdUrd (40 µM for both cell lines), or 10 mM hydroxyurea (HU) for the indicated times. Cell extracts were blotted for phospho-Ser317-Chk1 (P-Chk1), phospho-Thr68-Chk2 (P-Chk2), Chk1, or Chk2. TS, thymidylate synthase; TP, thymidine phosphorylase; UP, uridine phosphorylase; UK, uridine kinase; OPRT, orotate phosphoribosyltransferase; RR, ribonucleotide reductase; FUR, 5-fluorouridine; FUMP, 5-fluorouridine monophosphate; FUDP, 5-fluorouridine diphosphate; FUTP, 5-fluorouridine triphosphate; FdUMP, 5-fluorodeoxyuridine monophosphate; FdUDP, 5-fluorodeoxyuridine diphosphate; FdUTP, 5-fluorodeoxyuridine triphosphate.

5-fluorouracil has activity in multiple cancers and is one of the most widely prescribed anticancer agents, but its most frequent use is in colon cancer, where it is the basis for all modern colon cancer therapies. After uptake into cells, 5-FU undergoes complex metabolic reactions (Fig. 1A; rev. in [1]) to produce 3 known cytotoxic metabolites: FUTP (5-fluorouridine triphosphate), FdUMP (5-deoxyuridine monophosphate), and FdUTP (5-deoxyuridine triphosphate). The FUTP affects RNA metabolism following its incorporation into cellular RNA, where it disrupts snRNA, tRNA, and rRNA processing as well as the ribonucleolytic activity of the exosome and pseudouridylation of RNA [2]–[8].

In contrast, FdUMP and FdUTP disrupt DNA metabolism. These metabolites are produced following the conversion of 5-FU to FdUrd (5-fluorodeoxyuridine; floxuridine), which is also an FDA-approved chemotherapy agent for the treatment of colon cancer [9] and is often considered to have a similar mechanism of action as 5-FU. FdUrd is then phosphorylated to FdUMP and further phosphorylated to FdUTP [1]. The FdUMP inhibits thymidylate synthase (TS), which prevents the conversion of dUMP to dTMP, ultimately causing the depletion of dTTP, the accumulation of dUTP, and the disruption of dNTP ratios. In contrast, FdUTP, as well as the accumulated dUTP, are incorporated into DNA.

Consistent with their abilities to disrupt dNTP levels, both FdUrd and 5-FU activate the ATR checkpoint signaling pathway [10]–[17], a pathway that is triggered when DNA replication is inhibited and that also plays critical roles in promoting survival of cells experiencing replication stress produced by dNTP disruption and/or DNA lesions [18]. Once activated, ATR phosphorylates multiple substrates, including Chk1. The collective kinase activities of ATR and Chk1 orchestrate the S phase checkpoint and regulate DNA repair to promote cell viability and recovery [19]. Additionally, 5-FU and FdUrd also cause double strand DNA breaks [20], [21], which in turn activate the ATM checkpoint signaling pathway. The ATM pathway also regulates cell survival by either inducing apoptosis or preventing cell cycle progression and activating DNA repair, both of which promote survival [22]. Notably, however, it remains unclear whether the ATR and/or ATM checkpoint pathways play important roles in determining the survival of human colon cancer cells, the cells that are targeted by 5-FU and FdUrd in patients, when they are treated with these agents.

The uracil and 5-FU that are incorporated into the genome are also recognized by 2 DNA repair pathways that may play roles in the survival of cells treated with 5-FU and FdUrd. One pathway is the base excision repair (BER) pathway [1], [23]. In this pathway, genomically incorporated uracil and 5-FU are first recognized by uracil glycosylases, which excise the lesion, leaving an abasic site. The abasic site is further processed by an endonuclease (e.g., APE1), creating a single-stranded DNA break that is recognized by poly(ADP-ribose) polymerase, which poly(ADPribosyl)ates itself as well as other repair proteins, recruiting XRCC1 and other proteins that complete repair [24]. Investigations into the role of BER in cells treated with 5-FU or FdUrd have reached disparate conclusions using a wide variety of model systems. Given that these studies have shown that disabling BER protects, sensitizes, or has no effect on the cytotoxicity induced by 5-FU and FdUrd in these varied systems, including mouse [17], [23], [25]–[35], it remains unclear what, if any, role BER plays in the survival of colon cancer cells exposed to 5-FU or FdUrd.

The other implicated repair pathway is the mismatch repair (MMR) system. *In vitro* studies have found that the MMR pathway can remove 5-FU from artificial substrates containing 5-FU:G mispairs. Notably, however, studies in cells suggest that MMR plays only a minor role in the excision of 5-FU lesions in the genome [35]. Analyses of the effects of the MMR pathway on cell viability following treatment with 5-FU and FdUrd have generally indicated that cells with defective MMR are more resistant to 5-FU and FdUrd [10], [36]–[38], a result consistent with the finding that MMR-defective colon cancer patients do not benefit from 5-FU therapy [39].

Given the multiple mechanisms of action of 5-FU and FdUrd and the wide range of experimental systems used in these studies (including studies in mouse cell lines and in human cell lines derived from tumors that are not typically treated with 5-FU or FdUrd), we have initiated studies to address the roles of individual checkpoint signaling and DNA repair pathways in human cells derived from human tumors. In our first analysis, we found that 5-FU and FdUrd have very different mechanisms of action in ovarian cancer cells [17]. Disabling ATR or BER sensitized these cells to FdUrd, but not 5-FU, indicating that FdUrd kills cells by causing DNA damage and that 5-FU exerts its cytotoxicity by another mechanism, possibly by disrupting RNA metabolism. Notably, we also found that PARP inhibitors, which have shown unprecedented activity against ovarian tumors and other tumors that have mutated *BRCA1* and *BRCA2* [40]–[43], remarkably sensitized ovarian cancer cells to FdUrd but not 5-FU [17]. Given that FdUrd is active in ovarian cancer [44], [45], that defects in *BRCA1* and *BRCA2* (or other genes in the homologous repair pathway) are common in ovarian cancer [46], and that PARP inhibitors will likely have a role in the treatment of ovarian cancers [24], these findings suggested a novel therapeutic strategy in ovarian cancer. Notably, however, the biology of ovarian cancer is very different from the biology of colon cancer. The oncogenes and tumor suppressor genes commonly mutated in colon cancers (*APC, p53, PI3K, KRAS*) differ from the genes commonly mutated in ovarian cancers (*NF1, RB1, CDK12, CCNE1, NOTCH*) [46], [47]. Furthermore, the DNA repair pathways that are disrupted in colon cancer cells are vastly different from those disrupted in ovarian cancers. For example, the genes required for mismatch repair pathway (e.g., *MLH1* and *MSH2*) are often mutated or epigenetically silenced in colon cancers [39], [48], whereas defects in homologous recombination (e.g., *BRCA1* and *BRCA2* mutations) occur in ovarian cancers [46], [47]. Finally, ovarian and colon tumors have very different responses to 5-FU. Whereas 5-FU has very limited activity in ovarian cancer [49], [50], 5-FU is the cornerstone for all chemotherapy regimens used to treat colon cancers due to its high activity in this disease [1]. Therefore, given these biological differences between ovarian and colon cancers, and the fact that 5-FU is universally used in colon cancer chemotherapy regimens, we have now determined how 5-FU and FdUrd kill colon cancer cells to gain important insights underlying the biology of these agents and improve their use in the clinic to treat this disease. To that end, we initiated a systematic analysis of the roles of genotoxin-activated checkpoint signaling, the BER pathway, and the MMR pathway by depleting key signaling intermediates in these pathways using highly effective siRNAs. These findings not only further illuminate our understanding of the signaling and DNA repair pathways that are important in these cells, they also reveal that colon tumor cells are sensitized to FdUrd by small molecule PARP inhibitors that are currently in clinical trials, thus suggesting a novel therapeutic approach that combines FdUrd, a drug approved for the treatment of colon cancer, with a PARP inhibitor, an emerging class of agents with exciting anticancer activity.

## Materials and Methods

### Cell lines and culture

HT29, HCT-8, and HCT-116 cells were obtained from American Type Culture Collection. HCT-116.ch2 and HCT-116.ch3 [51] cells were from Scott Kaufmann (Mayo Clinic). Cells were cultured in RPMI-1640 media (Mediatech) supplemented with 10% fetal bovine serum (Atlanta Biologicals) at 37°C in 5% CO_2_. For clonogenic assays, the medium was supplemented with 100 U/mL penicillin and 100 µg/mL streptomycin (Mediatech).

### Materials

Reagents were from the following suppliers: 5-fluorouracil (APP Pharmaceuticals), FdUrd (Bedford Laboratories), ABT-888 (Selleck Chemicals and ChemieTek), AZD2281 (ChemieTek), KU-55933 (Tocris Bioscience), gemcitabine (Eli Lilly), SuperSignal Pico West (Thermo Scientific). All other materials were from Sigma-Aldrich.

Antibodies were as follows: phospho-Ser317-Chk1 (R&D Systems); phospho-Thr68-Chk2, ATR, horseradish peroxidase-linked rabbit IgG, and horseradish peroxidase-linked mouse IgG (Cell Signaling); Chk1 (Santa Cruz Biotechnology); Chk2 and ATM (Epitomics); APE1 (Abcam); XRCC1 (Bethyl Laboratories); beta-actin (Sigma-Aldrich); and HSP90, D. Toft (Mayo Clinic, Rochester, MN).

### Cell transfections and small interfering (si)RNAs

siRNAs were transfected by electroporation as described [17]. The transfected cells were cultured for 48 h before use. Sequences of siRNAs were: ATM-1, 5′-GCACCAGUCCAGUAUUGGC-3′ [52]; ATR-2, 5′-CCUCCGUGAUGUUGCUUGA-3′ [53]; XRCC1-2, 5′-CUCGACUCACUGUGCAGAA-3′ [54]; APE1, 5′-GGACAGAGCCAGAGGCCAA-3′; MLH1, 5′-GGAAGAUUCUGAUGUGGAA-3′; MSH2, 5′-GAUCCUAAUCUCAGUGAAU-3′; and luciferase, 5′-CUUACGCUGAGUACUUCGA-3′ [55].

### Clonogenic assays, cell lysis, and cell irradiation

Cell cycle analyses, clonogenic assays, cell lysis, immunoblotting and immunostaining were performed as described [56], [57]. For clonogenic assays using non-transfected cells, percent survivals of all individual and combination treatments were normalized to cells treated with vehicle only. For clonogenic assays using cells transfected with siRNA, percent survivals at each drug concentration were normalized to the vehicle-treated control for the given siRNA. Cells were irradiated with a RS-2000 Biological Irradiator, Rad Source (Suwanee, GA) 4–6 h after plating.

## Results

### 5-FU and FdUrd activate the ATR and ATM checkpoint signaling pathways in colon cancer cells

To assess the effects of 5-FU and FdUrd on the ATM and ATR checkpoint signaling pathways, we compared the abilities of these agents to activate checkpoint signaling in two colon cancer cell lines: HT29, which have a functional MMR system, and HCT-8, which have a mutations in *MSH6* and are MMR deficient [58]. Cells were treated with concentrations of each agent that inhibit clonogenicity by 90% (IC_90_) and, as a positive control, the replication inhibitor hydroxyurea. Activation of the ATM and ATR pathways was then assessed by immunoblotting for phosphorylated Chk1 and Chk2, two protein kinases that are phosphorylated and activated by ATR and ATM [59]. These studies revealed that 5-FU and FdUrd strongly activated Chk1 and Chk2 in HT29 cells (Fig. 1B), with 5-FU causing even greater levels of Chk1 phosphorylation than did FdUrd. Similarly, in HCT-8 cells, both agents induced Chk1 phosphorylation (Fig. 1C); however, in these cells 5-FU induced less Chk1 than did FdUrd. Analyses of Chk2 phosphorylation were not possible due to the very low levels of Chk2 in the HCT-8 cells (data not shown). Taken together, these results demonstrate that both agents cause genotoxic damage that activates checkpoint signaling pathways in colon cancer cells.

### ATR and ATM promote the survival of colon cancer cells treated with FdUrd but not 5-FU

**Figure 2:**
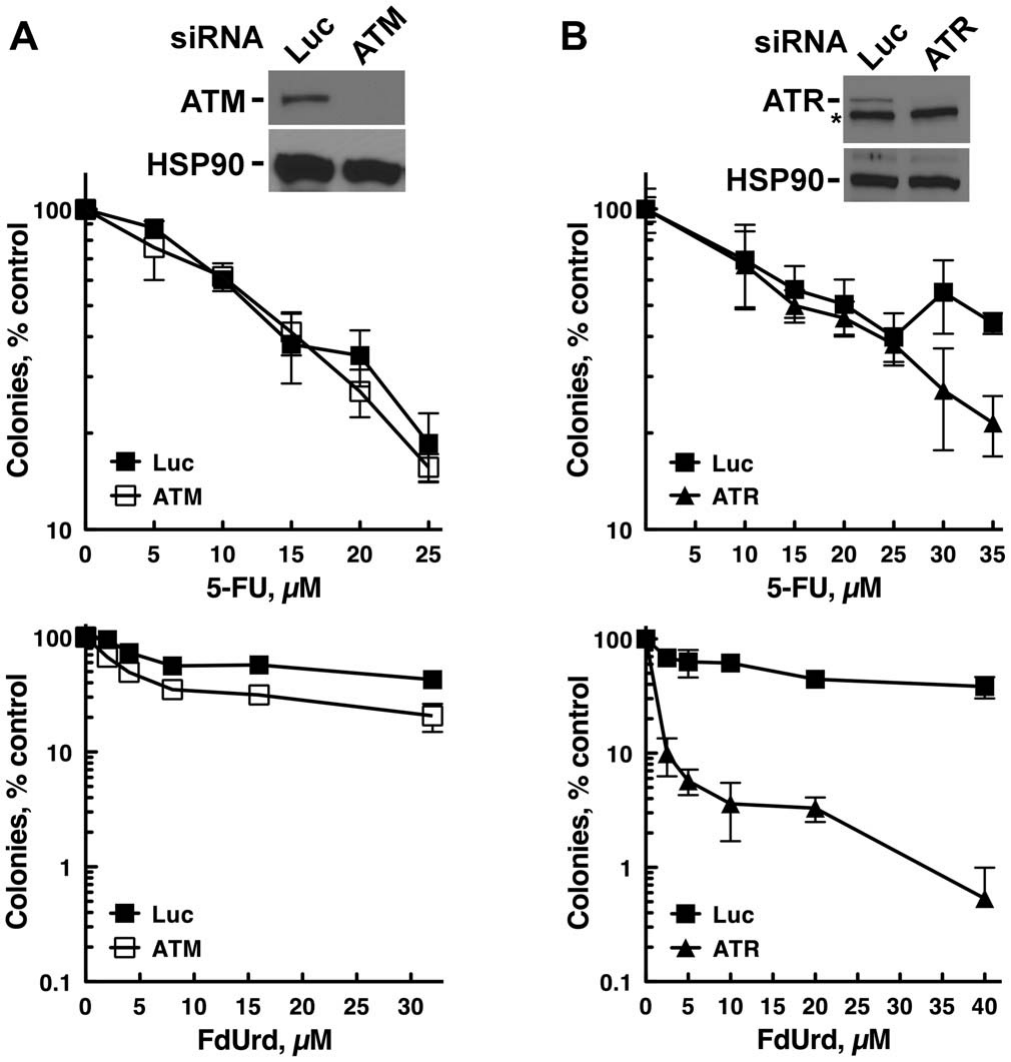
Depletion of ATR sensitizes to FdUrd but not 5-FU, whereas ATM depletion does not affect sensitivity to either agent. (A, B) HT29 cells, transfected with control (Luc), ATM (A), or ATR (B) siRNAs, were plated as single cells, exposed to the indicated concentrations of 5-FU or FdUrd for 24 h, washed, cultured for 10 d, and stained with Coomassie Blue. Transfected cells were also sequentially immunoblotted (insets) to detect ATM, ATR, and HSP90 (as a loading control). *, non-specific band.

**Figure 3:**
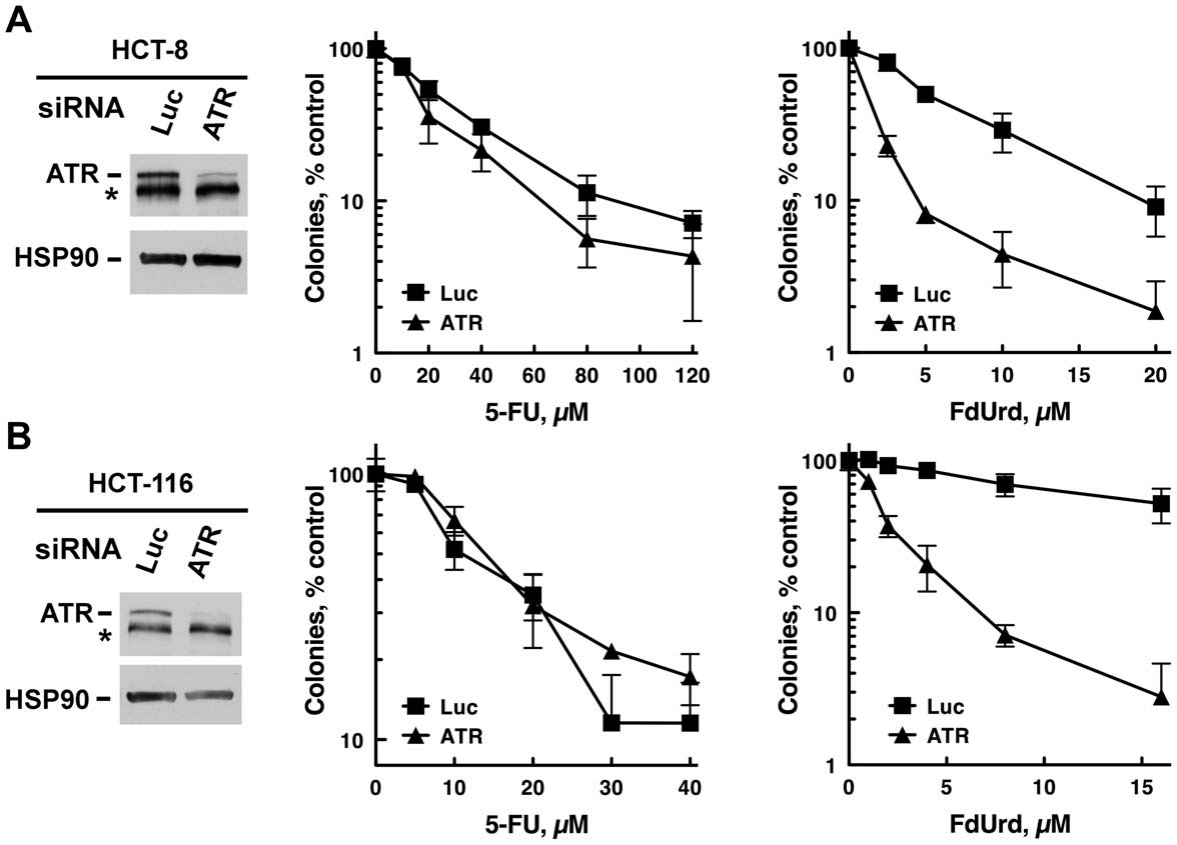
ATR depletion sensitizes HCT-8 and HCT-116 to FdUrd but not 5-FU. (A, B) HCT-8 (A) or HCT-116 (B) cells transfected with control (Luc) or ATR siRNAs were plated as single cells, exposed to the indicated concentrations of 5-FU or FdUrd for 24 h, washed, cultured for 10 d, and stained with Coomassie Blue. Transfected cells were also sequentially immunoblotted for ATR and HSP90 (a loading control). *, non-specific band.

ATM and ATR are the two apical kinase regulators of genotoxin-induced checkpoint signaling. To determine if either ATM or ATR activate pathways that affect the survival of cells treated with FdUrd or 5-FU, we transiently depleted ATM and ATR using siRNAs, and then assessed the capacity of cells treated with graded concentrations of FdUrd or 5-FU to proliferate using clonogenic assays. Surprisingly, depletion of ATM or ATR did not sensitize either cell line to 5-FU (Fig. 2A and 2B), even though this agent activated these pathways (see Fig. 1B and 1C). A far different picture emerged when the cells were treated with FdUrd. Depletion of ATR dramatically sensitized HT29 cells to FdUrd (Fig. 2B) and to gemcitabine (Fig. S1A), a nucleoside analog that inhibits ribonucleotide reductase and disrupts DNA replication when incorporated into DNA [60]. In contrast, ATM depletion (Fig. 2A) and the ATM inhibitor KU-55933 [61] (Fig. S1C), both of which sensitized to ionizing radiation (Fig. S1B and Fig S1D), had minimal effects on FdUrd cytotoxicity. Similar results were also seen in HCT-8 and HCT-116 cells, in which ATR depletion sensitized both cell lines to FdUrd but not 5-FU (Fig. 3).

### Disruption of BER by depleting XRCC1 sensitizes to FdUrd but not 5-FU

**Figure 4:**
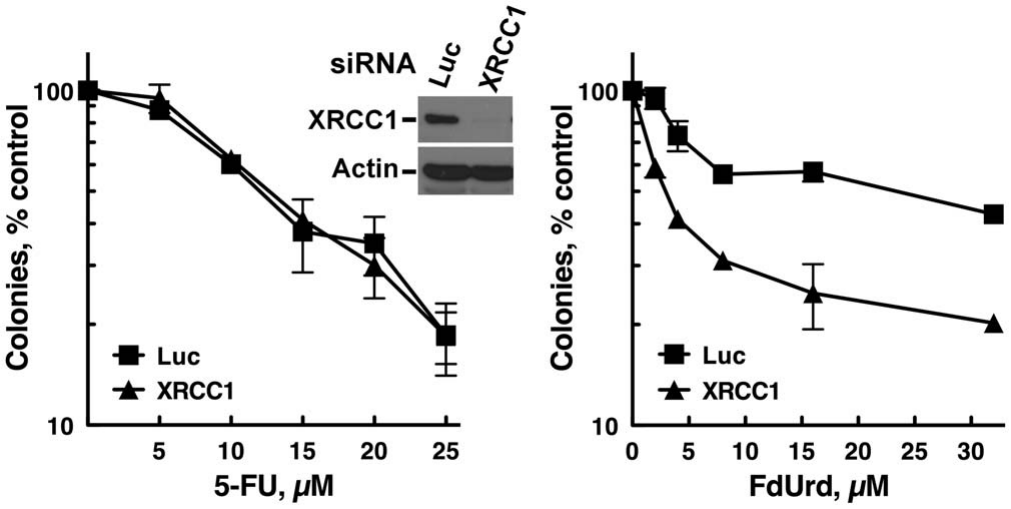
The BER pathway protects cells from FdUrd but not 5-FU. HT29 cells transfected with control (Luc) or XRCC1 siRNA were plated as single cells, exposed to the indicated concentrations of 5-FU or FdUrd for 24 h, washed, cultured for 10 d, and stained with Coomassie Blue. Transfected cells were also sequentially immunoblotted for XRCC1 and beta-actin (a loading control).

5-FU and FdUrd cause the accumulation of uracil and 5-fluorouracil in genomic DNA [23], [62]. Studies using purified uracil glycosylases have shown that synthetic substrates bearing uracil and 5-fluorouracil substituents are substrates for the BER machinery [35], [63]–[70]. Furthermore, a recent report demonstrated that in intact cells, uracil glycosylases remove 5-FU from the genomes of colon cancer cells exposed to FdUrd [35]; notably, however, in these studies, depletion of the glycosylases did not affect the sensitivity to FdUrd. Therefore, to examine whether disabling BER affected the sensitivity of HT29 cells to FdUrd, we used siRNAs to deplete XRCC1 and APE1, two downstream key participants in the BER pathway, and examined their sensitivity to FdUrd. Significantly, depletion of XRCC1 (Fig. 4) and APE1 (Fig. S2) sensitized cells to FdUrd. In contrast, XRCC1 depletion did not sensitize these cells to 5-FU (Fig. 4), thus indicating that BER does not play a role in promoting the survival of cells treated with 5-FU and further suggesting that 5-FU exerts its cytotoxic effects independently of DNA replication or damage.

### Small molecule PARP inhibitors sensitize colon cancer cells to FdUrd but not 5-FU

**Figure 5:**
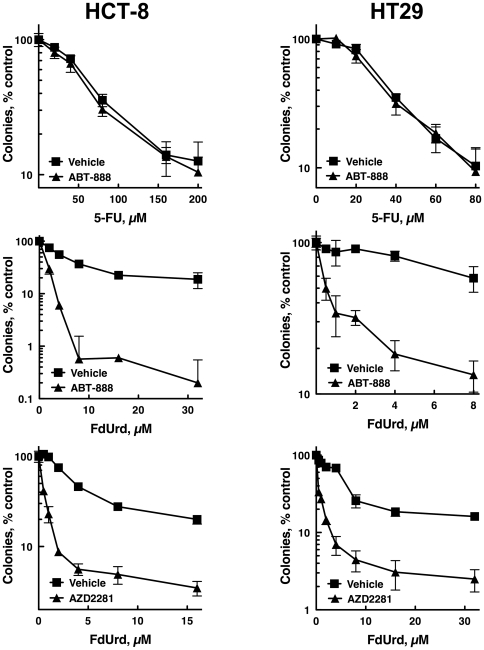
Small molecule PARP inhibitors sensitize HCT-8 and HT29 cells to FdUrd but not 5-FU. HCT-8 or HT29 cells were plated as single cells, exposed to the indicated concentrations of 5-FU or FdUrd along with 3 µM ABT-888, 300 nM AZD2281, or vehicle for 24 h. Following washing, 3 µM ABT-888 or 300 nM AZD2281 were re-added, and the cells were cultured for 10 d and stained with Coomassie Blue.

Given that XRCC1 and APE1 depletion sensitized colon cancer cells to FdUrd, and that PARP plays a key role in BER, we reasoned that PARP inhibitors may sensitize colon cancer cells to FdUrd. We therefore exposed HCT-8 and HT29 cells to graded concentrations of FdUrd or 5-FU along with 3 µM ABT-888 (veliparib [71]), a concentration that was reported previously to sensitize multiple tumor cell lines to a variety of chemotherapy agents [17], [72]. As shown in Fig. 5, ABT-888 robustly sensitized HCT-8 and HT29 cells to FdUrd, whereas ABT-888 did not alter the antiproliferative effects of 5-FU. To further demonstrate that PARP inhibitors sensitize these cells to FdUrd, we also tested the PARP inhibitor AZD2281 (olaparib [73]), which has shown unprecedented activity in heavily pretreated patients with BRCA1- and BRCA2-deficient tumors [40]–[43]. Similar to the results seen with ABT-888, AZD2281 robustly sensitized both cell lines to FdUrd (Fig. 5), further supporting the idea that PARP inhibition sensitizes colon tumor cells to FdUrd.

### Small molecule PARP inhibitor sensitization to FdUrd is independent of MMR status

**Figure 6:**
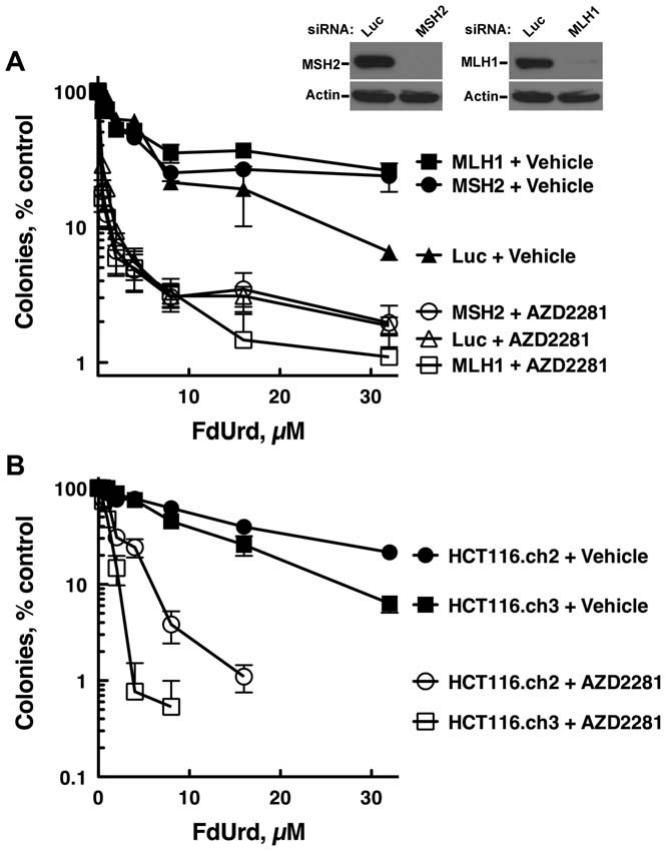
PARP inhibition sensitizes MMR-deficient cells to FdUrd. (A) HT29 cells transfected with control (Luc), MSH2, or MLH1 sRNAs were plated as single cells, exposed to the indicated concentrations of 5-FU or FdUrd for 24 h, washed, cultured for 10 d, and stained with Coomassie Blue. Transfected cells were also sequentially immunoblotted for MSH2 or MLH1 and beta-actin (a loading control). (B) HCT116.ch2 and HCT116.ch3 cells were exposed to the indicated concentrations of FdUrd along with vehicle or 300 nM AZD2281 for 24 h. Following washing, AZD2281 was re-added and the cells were cultured for 8 d to allow colony formation.

Previous reports demonstrated that cells with defects in MMR are more resistant to FdUrd [10], [36]–[38]. Similarly, patients treated with 5-FU do not benefit from 5-FU-based chemotherapies [39], suggesting that an intact MMR pathway promotes killing by 5-FU. Because combining FdUrd with a PARP inhibitor may be a potential therapeutic strategy, we reasoned that it would be important to determine whether tumor cells with defects in MMR, which occur in 15–20% of colon cancers [39], were sensitized to FdUrd by a PARP inhibitor. To assess how MMR status affects the sensitivity of colon cancer cells to FdUrd alone and to the combination of FdUrd plus AZD2281 we used two model systems. For the first model system, we used siRNAs to deplete MSH2 and MLH1. Both siRNAs were highly effective, causing near-complete loss of MLH1 and MSH2 (Fig. 6A) and disrupting MNNG (N-methyl-n′-nitro-n-nitrosoguanidine)-induced G2/M arrest (Fig. S3), which requires a functional MMR pathway [51]. Notably, HT29 cells depleted of MLH1 or MSH2 were severely sensitized to FdUrd by AZD2281, and were modestly resistant to FdUrd alone.

For the second model system, we employed the paired colon cells lines, HCT-116.ch2 and HCT-116.ch3 [51]. These cell lines were derived from parental HCT-116 cells, which have biallelic inactivating *MLH1* mutations that render them MMR-deficient [74]. The HCT-116.ch3 cells contain an additional chromosome 3, which encodes a functional MLH1 that restores MMR. The HCT-116.ch2 cells, which are used as a control, contain an additional chromosome 2 and like the parental cells are MMR-deficient. Consistent with previously published results, the MMR deficient HCT-116.ch2 cells were modestly more resistant to FdUrd than were the HCT-116.ch3 cells (Fig. 6B), which are MMR proficient [75]. Notably, however, AZD2281 robustly sensitized both cell lines to FdUrd. Taken together, these results demonstrate that colon cancer cells with defects in the MMR pathway can also be sensitized to FdUrd by a small molecule PARP inhibitor.

## Discussion

5-FU is among the most widely used anticancer chemotherapy agents, and it (or the 5-FU prodrug capecitabine) is the backbone of all chemotherapy regimes used to treat colon cancer [1], the third leading cause of cancer-related death in the United States [76]. Despite its widespread use in the treatment of colon cancer, it remains unclear how this agent kills colon tumor cells. Similarly, FdUrd, which is often considered to have a similar mechanism of action to 5-FU, is also used to treat colon tumors that have metastasized to the liver. To gain insight into how these agents affect colon cancer cells we first carried out comprehensive analyses of the roles of the ATM and ATR checkpoint signaling pathways in colon cancer cells exposed to 5-FU and FdUrd, and then analyzed the role of the BER pathway, a repair pathway that removes uracil and uracil analogs that are incorporated into the genome. We previously compared the mechanisms by which 5-FU and FdUrd kill ovarian cancer cells. Notably, however, 5-FU has very limited clinical activity against ovarian cancer [49], [50], and the DNA repair pathways that are disrupted in ovarian cancer differ from those disrupted in colon cancer. Specifically, ovarian cancers frequently exhibit “BRCAness” due to defects in *BRCA1* or *BRCA2*, or other ill-defined changes that disrupt the homologous recombination DNA repair pathway [46]. In contrast, in colon cancers the mismatch repair pathway is frequently mutated or silenced [39], [48], and the MMR pathway has been reported to affect cell killing by 5-FU and FdUrd [36]–[38], [77], [78]. Therefore, in the present report, we have performed head-to-head comparison of these agents in MMR-proficient and -deficient colon cancer cells that have been depleted of key checkpoint signaling and BER pathway intermediates. Importantly, these mechanistic studies have uncovered novel insights into how these agents kill colon cancer cells and identified a potential therapeutic strategy against colon cancer.

First, our studies demonstrated the ATR—but not the ATM—checkpoint signaling pathway plays a critical role facilitating the survival of cells treated with FdUrd. Although previous studies documented that FdUrd activates the ATM- and ATR-dependent checkpoints [10], [13], [79], these studies did not compare the effects of ATM and ATR depletions on the survival of tumor cells exposed to both agents. Here we have addressed that question. Surprisingly, we found that even though FdUrd has been reported to cause double-stranded DNA breaks [20], [21], ATM has only a minor role in FdUrd-induced killing. In contrast, ATR depletion severely sensitized to FdUrd, demonstrating that ATR plays a critical role in stabilizing stalled replication forks and preventing their collapse, thus promoting cell survival when cells are treated with replication inhibitors such as the nucleoside analog gemcitabine [60]. Therefore, the present studies suggest that the disruption of DNA replication that occurs when TS is inhibited and the subsequent disruption of dNTP levels is likely a major mechanism by which FdUrd causes cytotoxicity.

Second, the present results help clarify the role of BER in colon cancer cells exposed to 5-FU and FdUrd. Previous studies examining the role of the BER pathway have found disparate results, with increased, decreased, or unaltered sensitivity to 5-FU or FdUrd in a variety of experimental systems [17], [23], [25]–[35]. In contrast, the present results show that XRCC1 depletion sensitizes to FdUrd but not 5-FU. This finding, along with our published studies showing that an intact BER pathway protects ovarian cancer cells treated with FdUrd [17], indicates that FdUrd inflicts lesions that are cytotoxic to some human cancer cells. Consistent with these findings, two potent and highly specific small molecule inhibitors of PARP also sensitized to FdUrd. These results are similar to what was observed in ovarian cancer cells [17]. However, given that ovarian cancer cells often exhibit BRCAness (due to defects in homologous recombination) [46], [80], a phenotype that renders cells exquisitely sensitive to PARP inhibitors [81], it remained an unanswered question whether PARP inhibitors would also sensitize to FdUrd in colon cancer cells, which do not have defects in homologous recombination. It should be noted, however, that although our XRCC1 findings strongly support a protective role for BER, the effects of the PARP inhibitors may be more complicated. PARP not only plays an important role in BER but also participates in other DNA repair pathways and cell signaling pathways, raising the possibility that the tremendous sensitization seen with the PARP inhibitors may stem from effects on BER as well as other cellular pathways.

Third, the present studies show that depleting the apical regulators of checkpoint signaling (ATR and ATM) or disabling key BER pathway members (with XRCC1 and APE1 siRNAs or PARP inhibitors) did not sensitize to 5-FU. Such results strongly suggest that 5-FU is exerting its cytotoxic effects independently of its effects on DNA replication or integrity. Notably, this result is consistent with a number of studies showing that 5-FU mediates cell killing by incorporating into RNA and interfering with RNA metabolism [82]–[89]. In contrast, the finding that disabling the ATR and BER pathways strongly sensitizes to FdUrd, indicates that this agent kills colon tumor cells primarily by affecting DNA metabolism, thus demonstrating that 5-FU and FdUrd have very different mechanisms of action.

Finally, and most importantly, these studies, which were initiated to identify the checkpoint and DNA repair pathways that regulate colon tumor responses to FdUrd and 5-FU, demonstrated that BER was a critical repair pathway when these cells were exposed to FdUrd (but not 5-FU). Based on these findings, and the fact that PARP inhibitors disrupt BER, we then discovered that small molecule PARP inhibitors robustly sensitized MMR-deficient and –proficient colon cancer cells to FdUrd (but not 5-FU). These findings may be of particular importance in tumors with defects in MMR, which account for 15–20% of all colon cancers [39]. Previous studies found that MMR-deficient cell lines are less sensitive to 5-FU and FdUrd. Consistent with this result, clinical studies have shown that 5-FU has limited activity against MMR-deficient colon cancers compared to MMR-proficient tumors [39]. Given that 1) FdUrd is approved for the treatment of colon cancer; and 2) there are limited therapeutic options for these tumors because tumors with defects in MMR are commonly considered to be unresponsive to 5-FU-based therapies, our finding that PARP inhibitors robustly sensitize MMR-deficient cells to FdUrd raises the possibility that therapies that combine FdUrd with a PARP inhibitor may have activity against these tumors. Similarly, because PARP inhibitors also sensitize mismatch proficient tumors to FdUrd, this drug combination may also be useful in the treatment of these tumors. Further preclinical and clinical development of this combination is warranted.

## Supporting Information

Figure S1 **Effects of ATR and ATM disruptions on sensitivity to gemcitabine and ionizing radiation.** (A) ATR depletion sensitizes to gemcitabine. HT29 cells transfected with control (Luc) or ATR siRNAs from experiment shown in Fig. 2B were plated as single cells, exposed to the indicated concentrations of gemcitabine for 24 h, washed, and cultured for 10 d to allow colony formation. (B) ATM depletions sensitize to ionizing radiation (IR). HT29 cells transfected with control (Luc) or ATM siRNAs from experiment shown in Fig. 2A were plated as single cells, exposed to the indicated doses of ionizing radiation, and cultured for 10 d to allow colony formation. (C–D) The ATM inhibitor KU-55933 does not affect the sensitivity of HT-29 cells to FdUrd but sensitizes to ionizing radiation (IR). HT29 cells were plated as single cells and allowed to adhere for 4 h. For the FdUrd experiment (C), the cells were first exposed to the indicated concentrations of KU-55933 for 15 min and then FdUrd was added. Cells were then incubated for 24 h, washed, and cultured for 10 d to allow colony formation. For the IR experiment (D), the cells were exposed to the indicated concentrations of KU-55933 for 15 min, irradiated, washed after 24 h to remove the KU-55933, and cultured for 10 d to allow colony formation.
(TIF)

Figure S2 **APE1 depletion sensitizes HT29 cells to FdUrd.** Cells were transfected with control (Luc) or APE1 siRNAs. 48 h later, the cells were plated as single cells, treated with the indicated concentrations of FdUrd for 24 h, washed, and cultured for 10 d to allow colony formation.
(TIF)

Figure S3 **Depletion of MSH2 and MLH1 disrupts MNNG-induced G2/M cell cycle arrest.** HT29 cells transfected with control (Luc), MSH2, or MLH1 siRNAs were incubated with 3 µM N-methyl-N′-nitro-N-nitrosoguanidine (MNNG) for 24 h, stained with propidium iodide and analyzed by flow cytometry for DNA content.
(TIF)
