# Supplementary figures and images for: Checkpoint Signaling, Base Excision Repair, and PARP Promote Survival of Colon Cancer Cells Treated with 5-Fluorodeoxyuridine but Not 5-Fluorouracil
Source: PLoS One. 2011 Dec 15;6(12):e28862. doi: 10.1371/journal.pone.0028862 (PMC3240632; doi:10.1371/journal.pone.0028862)

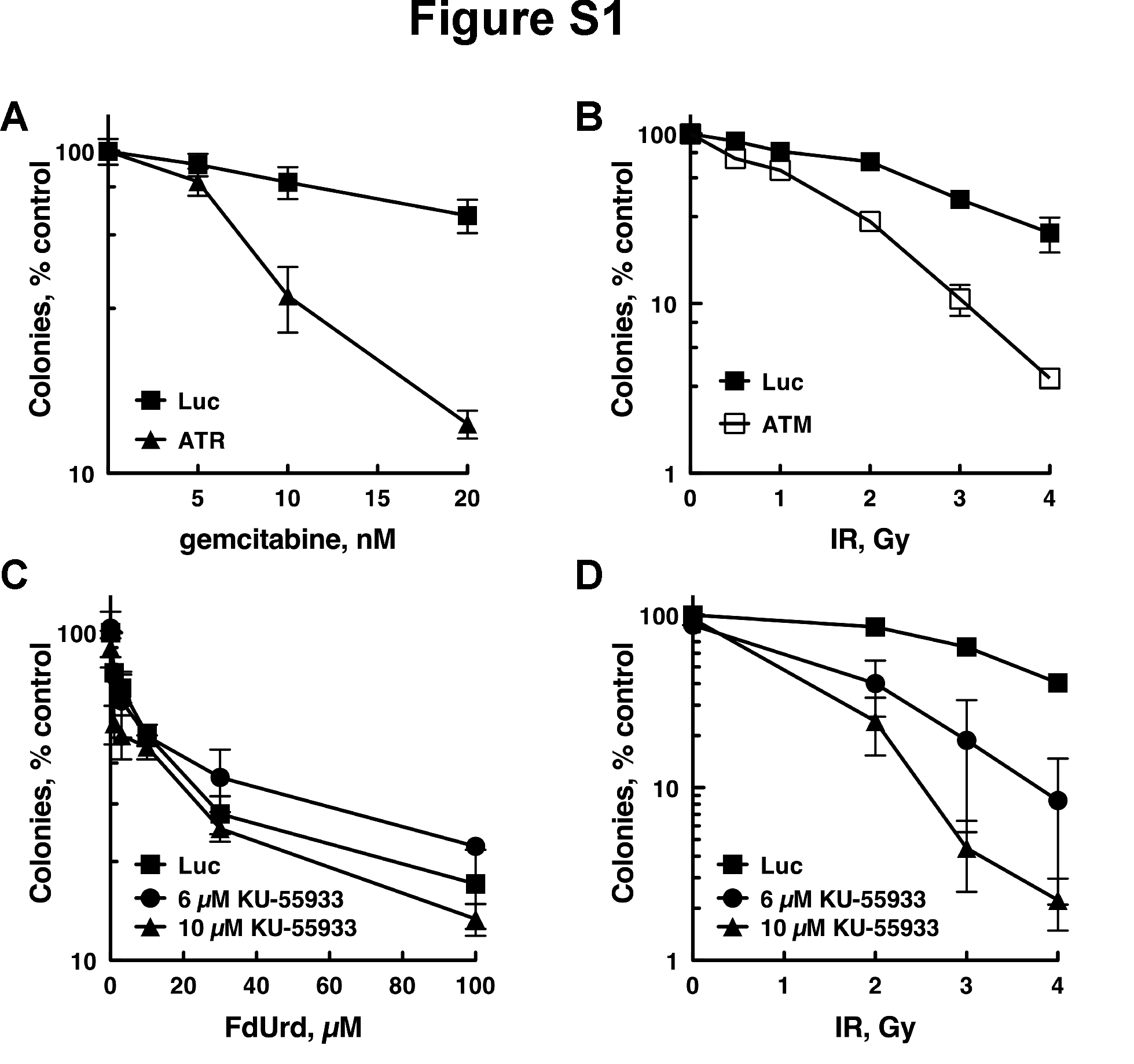

Supplement: Figure S1 — Effects of ATR and ATM disruptions on sensitivity to gemcitabine and ionizing radiation. (A) ATR depletion sensitizes to gemcitabine. HT29 cells transfected with control (Luc) or ATR siRNAs from experiment shown in Fig. 2B were plated as single cells, exposed to the indicated concentrations of gemcitabine for 24 h, washed, and cultured for 10 d to allow colony formation. (B) ATM depletions sensitize to ionizing radiation (IR). HT29 cells transfected with control (Luc) or ATM siRNAs from experiment shown in Fig. 2A were plated as single cells, exposed to the indicated doses of ionizing radiation, and cultured for 10 d to allow colony formation. (C–D) The ATM inhibitor KU-55933 does not affect the sensitivity of HT-29 cells to FdUrd but sensitizes to ionizing radiation (IR). HT29 cells were plated as single cells and allowed to adhere for 4 h. For the FdUrd experiment (C), the cells were first exposed to the indicated concentrations of KU-55933 for 15 min and then FdUrd was added. Cells were then incubated for 24 h, washed, and cultured for 10 d to allow colony formation. For the IR experiment (D), the cells were exposed to the indicated concentrations of KU-55933 for 15 min, irradiated, washed after 24 h to remove the KU-55933, and cultured for 10 d to allow colony formation. (TIF) [file pone.0028862.s001.tif]

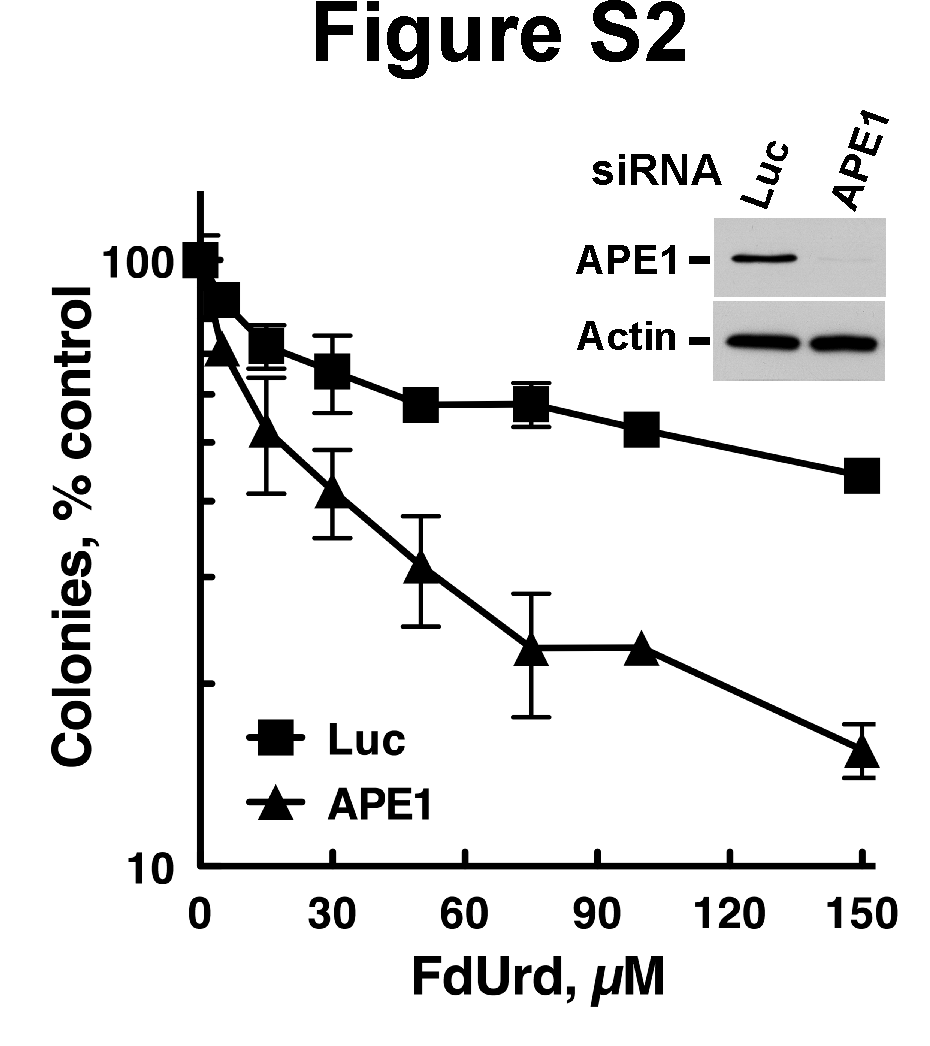

Supplement: Figure S2 — APE1 depletion sensitizes HT29 cells to FdUrd. Cells were transfected with control (Luc) or APE1 siRNAs. 48 h later, the cells were plated as single cells, treated with the indicated concentrations of FdUrd for 24 h, washed, and cultured for 10 d to allow colony formation. (TIF) [file pone.0028862.s002.tif]

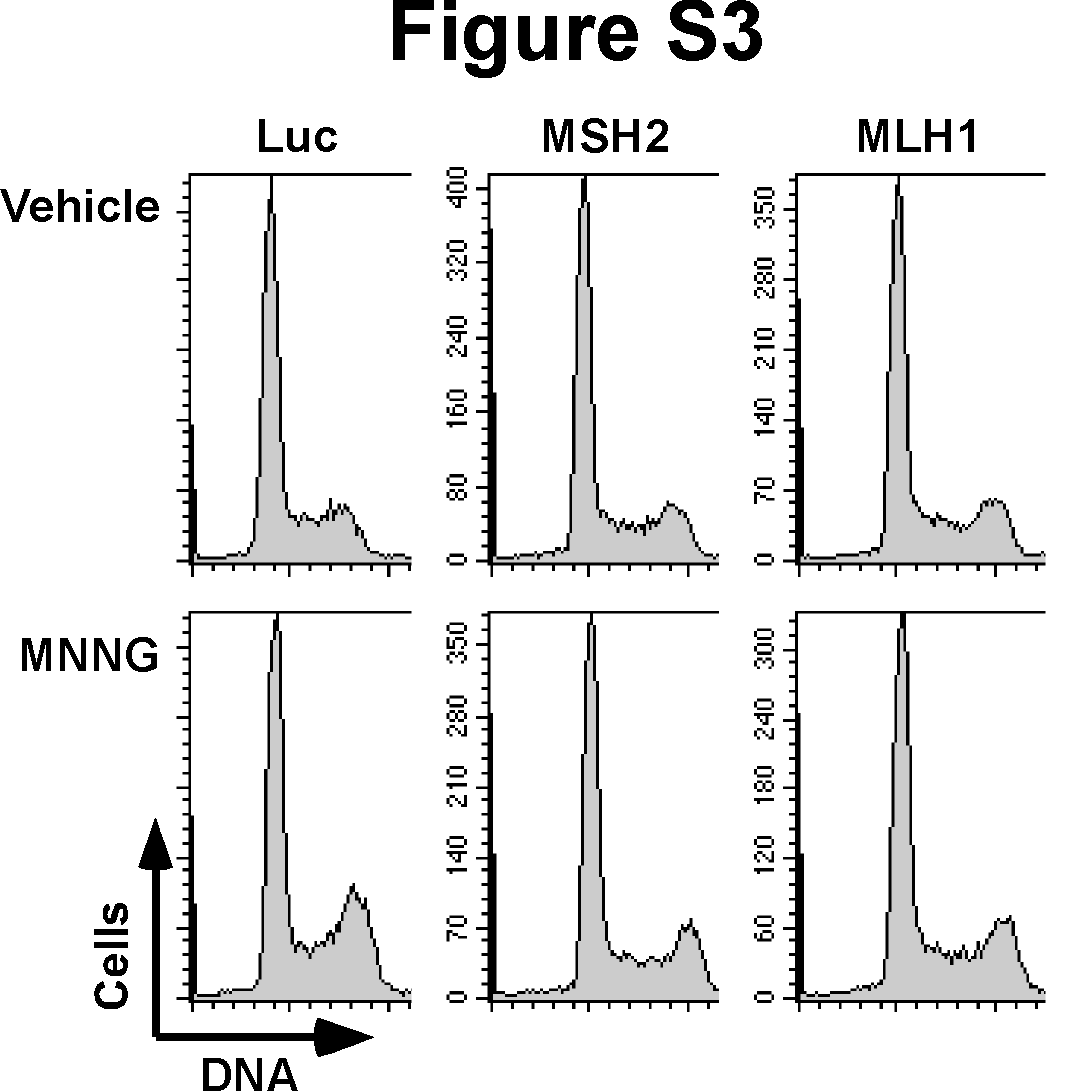

Supplement: Figure S3 — Depletion of MSH2 and MLH1 disrupts MNNG-induced G2/M cell cycle arrest. HT29 cells transfected with control (Luc), MSH2, or MLH1 siRNAs were incubated with 3 µM N-methyl-N′-nitro-N-nitrosoguanidine (MNNG) for 24 h, stained with propidium iodide and analyzed by flow cytometry for DNA content. (TIF) [file pone.0028862.s003.tif]
